# The Protective Effects of Bushen Daozhuo Granule on Chronic Non-bacterial Prostatitis

**DOI:** 10.3389/fphar.2023.1281002

**Published:** 2024-01-04

**Authors:** Dalin Sun, Dong Xing, Dandan Wang, Yuanyuan Liu, Bin Cai, Weimin Deng, Qinglin Hu, Wenjun Ma, Baofang Jin

**Affiliations:** ^1^ Andrology Department of Integrative Medicine, Zhongda Hospital, School of Medicine, Southeast University, Nanjing, China; ^2^ School of Medicine, Southeast University, Nanjing, China; ^3^ Department of Urology, Chuzhou Integrated Hospital of Chinese and Western Medicine, Affiliated to Anhui University of Traditional Chinese Medicine, Chuzhou, China

**Keywords:** Bushen Daozhuo granule (BSDZG), chronic non-bacterial prostatitis (CNP), anti-inflammation, anti-apoptosis, TNF-α

## Abstract

**Background:** Chronic non-bacterial prostatitis (CNP), one of the most common chronic diseases in urology, leads to pain in the prostate and dysuria, critically affecting the physical or mental health of patients. However, there are no standard treatment approaches for the treatment of CNP in the clinic. Although the clinical application of Bushen Daozhuo granule (BSDZG) offers hope to CNP patients in China, the mechanisms of BSDZG in treating CNP are still not entirely clear. Hence, we aimed to investigate the novel therapeutic mechanisms of BSDZG on CNP.

**Methods:** In this study, we first assayed the prostate index of rats and then determined the anti-inflammatory and anti-apoptotic effects of BSDZG on CNP *in vivo* and *in vitro* by employing ELISA kits and TUNEL staining. Next, we investigated whether the anti-inflammatory and anti-apoptotic mechanisms of BSDZG on prostate protein-induced rats and lipopolysaccharide (LPS) induced RWPE-1 cells were related to the AKT, p38 MAPK, and NF-κB pathways with the help of Western blot. Finally, the influence of BSDZG on the interaction between the p38 MAPK and NF-κB pathway in LPS-induced RWPE-1 cells was explored by adopting dehydrocorydaline (DHC, p38 MAPK activator) with the help of ELISA kits and Western blot.

**Results:** *In vivo*, BSDZG effectively reduced the prostate index. *In vivo* and *in vitro*, BSDZG dramatically declined the level of two pro-inflammatory cytokines, TNF-α and IL-1β, as well as the apoptosis rate. Moreover, *in vivo* and *in vitro*, BSDZG memorably upregulated the expression level of p-AKT, and substantially downregulated the expression level of p-p38 MAPK and NF-κB2. The activation of p38 MAPK significantly reversed the moderation effects of BSDZG on the level of TNF-α and IL-1β, as well as the expression level of p-p38 MAPK and NF-κB2 *in vitro*.

**Conclusion:** To sum up, the *in vivo* and *in vitro* therapeutic mechanisms of BSDZG on CNP were reflected as the anti-inflammation and anti-apoptosis that was formed by inhibiting the level of pro-inflammatory cytokines, TNF-α and IL-1β, to regulate the AKT, p38 MAPK, and NF-κB pathways, and the anti-inflammatory effect of BSDZG was realized by suppressing the p38 MAPK pathway to inhibit the downstream NF-κB pathway.

## Introduction

Prostatitis refers to varying degrees of pathological changes in prostate tissues induced by pathogens or some non-infectious factors, which is a kind of disorder with characteristics of discomfort or even pain in the pelvic area as well as abnormal urination in the clinic (He, Luo et al., 2023). According to the standardized classification approach proposed by World Health Organization, the Meares-Stamey 4-glass test, prostatitis is divided into four kinds based on the difference in the number of leukocytes and the kind of bacterium in voided bladder one, voided bladder two, voided bladder three, and expressed prostatic secretion, including acute bacterial prostatitis, chronic bacterial prostatitis, chronic non-bacterial prostatitis (CNP), and prostatodynia (Drach, Fair et al., 1978). CNP is the most common clinical disease among them, accounting for more than 90% of prostatitis (Park, Park et al., 2019). The pathogenesis of CNP is complex, mainly including pathogen infection, immune abnormality, psychological disorders, urinary reflux, endocrine abnormality, epithelial dysfunction of the lower urinary tract, and abnormal activity of the pelvic floor neuromuscular (Batstone, Doble et al., 2002; Dellabella, Milanese et al., 2006; Parsons, 2007; Anderson, Orenberg et al., 2008; Shoskes, Berger et al., 2008; Hou, Long et al., 2012; Riegel, Bruenahl et al., 2014). The treatment principle of CNP is individual based on the patient’s symptoms, which is aimed at alleviating clinical symptoms, such as pain, dysuria, and mental or psychological disorders (Liu, Liu et al., 2021). However, the overall treatment effect on CNP is unsatisfactory in the clinic, which is usually accompanied by a high recurrence rate (Chen, Wu et al., 2011). Therefore, it is urgent to investigate a therapeutic approach to provide application prospects for the clinical treatment of CNP.

Although CNP is not induced by bacteria, CNP will further lead to diverse pathologic changes, including gland atrophy, interstitial fibrosis, and inflammatory infiltration (e.g., neutrophils, lymphocytes, and monocytes) (Kang, Park et al., 2019). Moreover, there were several studies also proved that signaling pathways related to regulating inflammation and apoptosis, especially for the nuclear factor kappa-B (NF-κB), p38 mitogen-activated protein kinase (p38 MAPK), and protein kinase B (AKT) pathways, possessed a prominent correlation with the onset and prognosis of chronic prostatitis (Feng, Dong et al., 2021; Wang, Li et al., 2023; Yang, Zhang et al., 2023). Therefore, we reasonably speculate that the regulation of apoptosis-related pathways and inflammatory reactions is expected to be the novel therapeutic approach to CNP. Out of the characteristics of multi-components and multi-targets, traditional Chinese medicine may show extraordinary talents in the therapy of CNP. As previous studies reported, multiple Chinese medicinal compound formulae have been affirmed to improve CNP predominantly through regulating inflammatory responses (Zang, Tian et al., 2021; Yi, Pan et al., 2022; Yunpeng, Wentao et al., 2023). However, there were few reports about the mechanism of traditional Chinese medicine in curing CNP from both anti-inflammatory and anti-apoptotic perspectives.

Bushen Daozhuo granule (BSDZG), one of the applied medications for CNP in China that has been confirmed to contain hyperoside and schisandrin by our previous study, is composed of eleven kinds of Chinese materia medica, most of which have been demonstrated to possess anti-inflammation and anti-apoptosis activities, especially for neuroinflammation and renal-related inflammation (Han, Pang et al., 2020; Zhong, Qiu et al., 2020; Cai, Wang et al., 2021; Song, Chen et al., 2021; Zhu, Dong et al., 2021; Kwak, Park et al., 2022; Han, Lee et al., 2023; Hou, Yang et al., 2023; Hu, Liu et al., 2023). Accordingly, in this paper, we aimed to elucidate the mechanism of BSDZG in treating CNP via anti-inflammation and anti-apoptosis from *in vivo* and *in vitro* experiments.

## Materials and Methods

### Drugs and reagents

BSDZG was provided by Tianjiang Pharmaceutical Co., Ltd. (Jiangsu, China). Bicinchoninic acid (BCA) kit and, complete Freund’s adjuvant, and physiological saline were phrased from Beyotime Biotechnology Co., Ltd. (Shanghai, China). H&E staining kit and TUNEL assay kit were bought from Abcam (Cambridge, UK). Dehydrocorydaline (DHC) and dimethyl sulfoxide (DMSO) were obtained from MedChemExpress (New Jersey, USA). Keratinocyte-SFM medium was phrased from Thermo Fisher Scientific Inc. (Massachusetts, USA). Fetal bovine serum (FBS) and trypsin were obtained from Biological Industries (Beit Haemek, Israel). Lipopolysaccharide (LPS) was brought from Aladdin Biochemical Technology Co., Ltd. (Shanghai, China). Tumor necrosis factor-α (TNF-α) ELISA assay kit and interleukin-1β (IL-1β) ELISA assay kit were brought from Meibiao Biology (Nanjing, China). DAPI solution, anti-AKT, anti-phosphorylated AKT (p-AKT), anti-p38 MAPK, anti-phosphorylated p38 MAPK (p-p38 MAPK), anti-Bax, ani-Bcl-2, anti-NF-κB2, anti-TNF-α, anti-tumor necrosis factor receptor (TNFR), anti-GAPDH, and anti-vinculin were phrased from Servicebio Technology (Wuhan, China).

### Preparation of BSDZG

A total of 7.5 g BSDZG purchased from Tianjiang Pharmaceutical Co., Ltd. (Jiangsu, China) was composed of 7.1% *Cuscuta chinensis* Lam., 7.1% *Dioscorea hypoglauca* Palibin, 7.1% *Schisandra chinensis* (Turcz.) Baill., 7.1% *Plantago asiatica* L., 7.1% *Alpinia oxyphylla* Miq., 7.1% *Lindera aggregata* (Sims) Kosterm., 14.3% *Ostrea gigas* Thunberg., 7.1% *Acorus calamus* L., 14.3% *Verbena officinalis* L., 7.1% *Hirudo nipponica*, and 14.3% *Vaccaria segetalis* (Neck.) Garck, which was equivalent to 140 g of the crude drug. BSDZG content was extracted with 60 mL of distilled water, by boiling at 100°C for 20 min. Subsequently, the extract was dissolved in normal saline to form a suspension of 0.15 g/mL, which was stored at 4°C for further use. By integrating the recommended dosage conversion ratio between rats and humans determined by expert opinion based on consideration of previously observed clinical treatment effects, the animal experimentation protocol was established with dosages of 0.77 g/kg/day and 1.54 g/kg/day.

### Rats and establishment of the animal model

A total of 60 male Wistar rats (weighing 330–380 g) at 10 weeks old purchased from Beijing Charles River Experimental Animal Technology Co., Ltd. were adaptively fed for 1 week in the condition with a temperature of 21°C ± 2°C and a humidity of 40%–70%. The animal study was approved by the Animal Care and Use Committee of Southeast University with ethical approval numbered: 20211210032. The *in vivo* CNP model adopted in the paper that drew lessons from a previous study was established by the purification solution of rats’ prostate protein supplemented with an immunologic adjuvant (Yang, Meng et al., 2019). In short, the overall operation processes were as follows. Firstly, prostate tissues from rats were harvested and prepared into supernatants that were centrifuged (10,000 g, 30 min) from the prostate tissue homogenate. Then, the protein content in supernatants was detected by BCA kits and was diluted into 40 mg/mL to obtain the prostate protein purification solution. Next, the prostate protein purification solution was mixed with complete Freund’s adjuvant (50:50, v/v) to form an emulsified immune agent. Finally, the emulsified immune agent (1 mL) was subcutaneously multipoint (100 μL per point) injected into the back of rats once per 15 days for four times.

## Animal experiment protocol

All rats were randomly divided into four groups (*n* = 15) as follows, control group, model group, low dose of BSDZG (L-BSDZG) group, and high dose of BSDZG (H-BSDZG) group. At day 0, day 15, day 30, and day 45 after 1-week adaptive feeding, apart from rats in the control group who were injected in multiple spots (100 μL per point) with physiological saline (1 mL, hypodermic injection, i. h.), rats in the other three groups all respectively suffered the multipoint (100 μL per point) injection of the emulsified immune agent (1 mL, i. h.). Rats in the control group and model group were treated with physiological saline (10 mL/kg, oral gavage, i. g.) once per day for 30 days from day 46. According to the equivalent dose ratio between humans and rats based on body surface area conversion that is widely acknowledged, the administered dosage of BSDZG employed in this paper was equivalently converted from the clinical dosage, namely, the dosage of BSDZG for animal experiments was 6.17 times of clinical dosage (Wojcikowski and Gobe, 2014). Rats in the L-BSDZG group and H-BSDZG group were respectively administered with 0.77 g/kg and 1.54 g/kg (i.g.) suspension of BSDZG dissolved in the physiological saline once per day for 30 days from day 46.

### Prostate index assay

At 1 h after the final administration, all rats were anesthetized with isoflurane and executed. The prostate was isolated and weighed for the calculation of the prostate index. The prostate index was generated by dividing the weight of the prostate by the weight of the rat.

### H&E staining

The fixed prostate in 4% paraformaldehyde was embedded into paraffin wax. The prostate tissue was sliced into 4 μm-thick sections. After dewaxing, tissue sections were first dyed with hematoxylin solution for 5 min, then counter-stained by eosin for 2 min, and finally visualized by the optical microscope.

### Preparation of BSDZG-containing serum

The grouping and administration method of rats for the preparation of BSDZG-containing serum was the same as those for treating CNP rats, accompanied by the only difference that the administered duration for the former was 3 days. The blood was collected from the abdominal aorta of rats anesthetized by halothane at 1 h after the last administration. The gathered blood was first subjected to room temperature for 2 h and then was centrifuged (3,000 g, 10 min) at 4°C to separate BSDZG-containing serum. The obtained serum was firstly inactivated under the condition of 56°C water bath, and then filtered by 0.22 μm sterile microporous filter membrane in the sterile ultra-clean bench, finally sub-packaged and stored at −20°C until use. During the cellular experiments, the blank serum (BS), L-BSDZG serum, and H-BSDZG serum were diluted 10 times by culture medium.

### Cell culture and establishment of cellular model

RWPE-1, the human prostate epithelial cell line, was purchased from the cell bank of the Chinese Academy of Sciences, and cultured in Keratinocyte-SFM medium supplemented with 100 mg/mL penicillin/streptomycin and 10% FBS (Ueda, Kono et al., 2022). The cultured medium was exchanged every 2 days. According to the previous study, LPS (10 μg/mL) was subjected to RWPE-1 cells for 24 h to induce the CNP model *in vitro* in the present study (Song, Chen et al., 2021). The RWPE-1 cells at the logarithmic growth period were digested by trypsin and centrifuged for passage.

### Cellular experiment protocol

For the research on the effect of BSDZG on the cellular CNP model, RWPE-1 cells at the logarithmic growth period were divided into five groups as follows, normal control (NC) group, LPS group, LPS + BS group, LPS + L-BSDZG group, LPS + H-BSDZG group. The normal cultivation conditions were adopted in the NC group. The normal cultivation conditions additionally supplemented by LPS were employed in the LPS group. The 10% FBS was replaced by 10% BS that was respectively replenished by LPS, L-BSDZG serum, and H-BSDZG serum in the LPS + BS group, LPS + L-BSDZG serum group, and LPS + H-BSDZG serum group. For the research on the effect of BSDZG on the p38 MAPK signaling pathway in the cellular CNP model, RWPE-1 cells at the logarithmic growth period were divided into five groups as follows, LPS group, LPS + DMSO group, LPS + DHC group, LPS + H-BSDZG group, LPS + H-BSDZG + DHC group. The combination of 10% BS and LPS was applied in the LPS group. The difference between the LPS + DMSO group and the LPS + DHC group with the LPS group was that DMSO (20 μmol/mL, vehicle of DHC) or DHC (5 mol/L, p38 MAPK activator) was additionally added into the combination of 10% BS and LPS. The difference between the LPS + DMSO group and the LPS + DHC group with LPS + H-BSDZG group and LPS + H-BSDZG + DHC group was that 10% BS was replaced by H-BSDZG serum.

### Pro-inflammatory cytokines detection

The level of pro-inflammatory cytokines in the prostate tissue and cellular supernatant, including TNF-α and IL-1β, was detected by respective ELISA kits in line with the corresponding producer’s instructions.

### TUNEL staining

The apoptosis rate *in vivo* and *in vitro* was measured by using TUNEL staining. For the detection of apoptosis rate *in vivo*, the pretreatment processes of TUNEL staining were the same as those of H&E staining. For the detection of apoptosis rate *in vitro*, RWPE-1 cells were first seeded into 6-well plates for 24 h and suffered from corresponding treatments. The subsequent *in vivo* and *in vitro* stained operations were the same. Proteinase K solution (20 μg/mL) was first added to the tissue sections and cells for 20 min at room temperature after dewaxing. Then, 100 μL 1× Equilibration Buffer that was diluted by ddH2O was incubated with tissue sections and cells for 20 min at room temperature. Next, 50 μL TdT buffer was subjected to tissue sections and cells for further incubation of 60 min at 37°C. Finally, DAPI (2 μg/mL) was adopted for counterstain. Tissue sections and cells were visualized by employing the fluorescence microscope at 460 nm and 620 nm, respectively. The number of red-stained and blue-stained cells was counted with the help of ImageJ software. The apoptosis rate = (number of positive cells/total number of cells) × 100%.

### Western blot

Total proteins were extracted from the prostate tissue and RWPE-1 cells in each group by adopting RIPA lysis solution. After the concentration of total proteins was determined by employing BCA kits, they were denatured by boiling water. Total proteins were separated with the help of SDS-PAGE before target proteins were transferred to the PVDF membrane. For *in vivo* and *in vitro* studies, the PVDF membranes contained target proteins were incubated with multiple primary antibodies for one night at 4°C, including AKT (1: 1000), p-AKT (1: 1000), p38 MAPK (1: 1000), p-p38 MAPK (1: 1000), Bax (1: 1000), Bcl-2 (1: 1000), NF-κB2 (1: 1000), TNF-α (1: 1000), TNFR (1: 1000), GAPDH (1: 1000), and vinculin (1: 1000). The corresponding secondary antibodies were subsequently incubated with PVDF membranes for 30 min at room temperature. Finally, ImageJ software was used to analyze the grayscale value of all target protein strips that were stained by a hypersensitive ECL chemiluminescence kit and then observed by the Western blot imaging system.

### Statistical analysis

All experimental data were analyzed and transformed into figures in the form of mean ± standard deviation by adopting GraphPad Prism 7.0 (La Jolla, CA. USA). The differences between groups were evaluated by applying a one-way analysis of variance. *p* < 0.05 means significant statistical differences.

## Results

### Effects of BSDZG on the prostate index of rats

**FIGURE 1:**
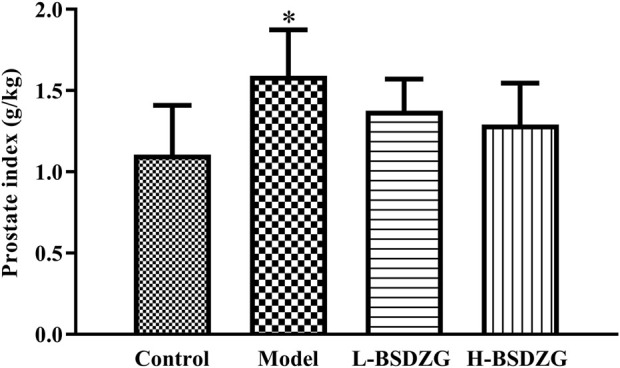
Effects of BSDZG on prostate index. ^*^ *p* < 0.05, compared with the control group.

The prostate index, one basic indicator that reflects inflammation in the prostate, not only reflects organ atrophy and degenerative changes but also generally implies the occurrence and development of inflammation. As Figure 1 shows, the prostate index of rats in the model group was significantly higher than that in the control group (*p* < 0.05). Although the prostate index of rats in the L-BSDZG group and the H-BSDZG group was lower than that in the model group, the difference was not statistically significant (*p* > 0.05). The above results indicated that BSDZG possesses the prospective anti-inflammation effects on rats with CNP.

### Effects of BSDZG on pathological changes of the prostate tissue of rats

**FIGURE 2:**
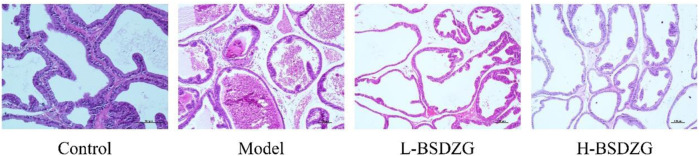
Representative H&E staining images of effects of BSDZG on pathological changes of the prostate tissue.

H&E staining can reflect the most intuitive manifestation of the degree of inflammati**on** of the pathological changes of the prostate tissue. Illustrated in Figure 2, compared with the control group, the pathological changes in the model group were mainly manifested as a partial destruction of the acinar structure, reduction of intraluminal folds, apparent swelling of the interstitial tissue, and obvious infiltration of inflammatory cells. Compared with the model group, the changing trend of acinar structure and intraluminal folds was reversed, and the swelling of interstitial tissue as well as the infiltration of inflammatory cells were reduced in the L-BSDZG group and the H-BSDZG group.

### Effects of BSDZG on pro-inflammatory cytokines and related proteins of the prostate tissue of rats

**FIGURE 3:**
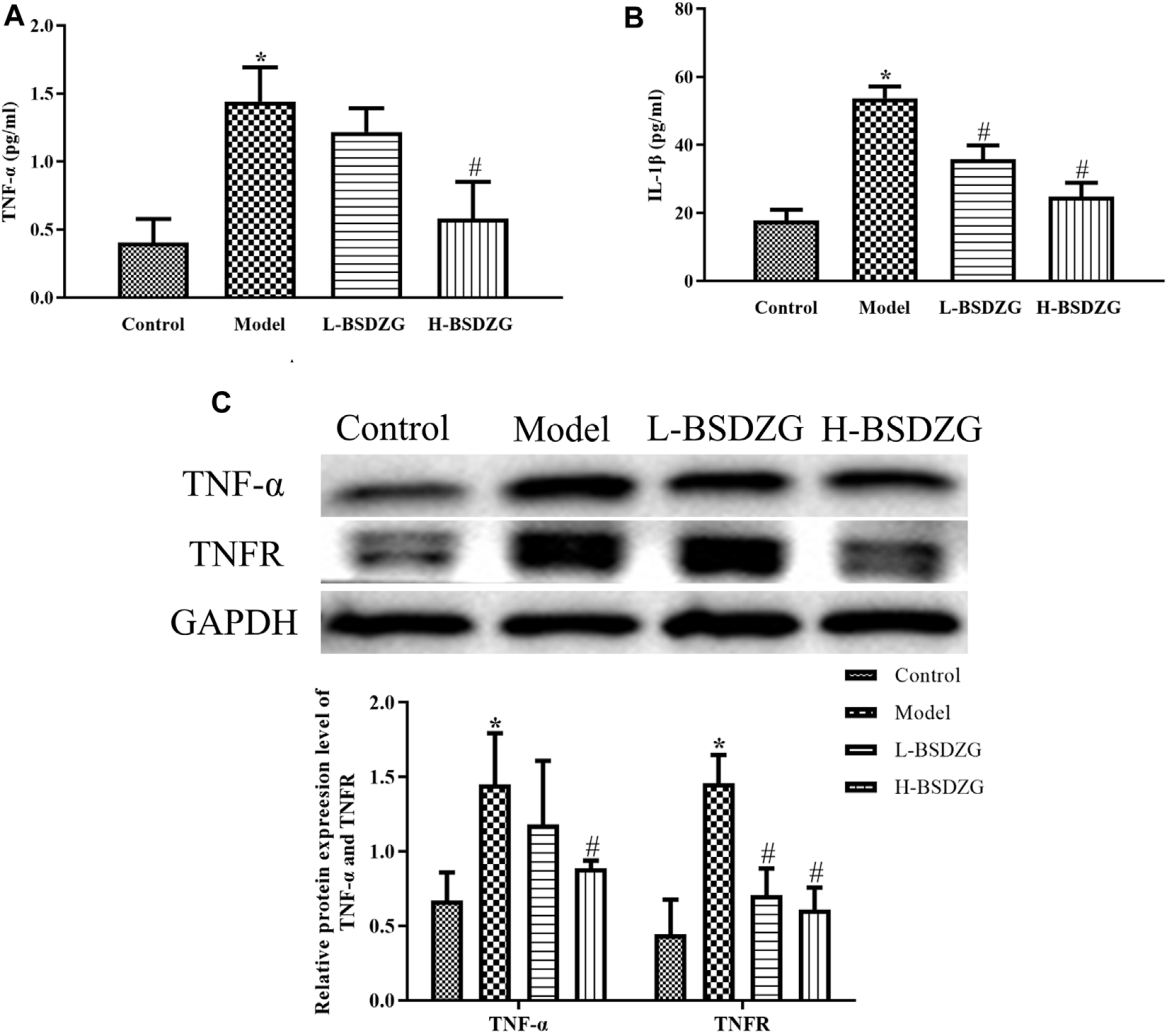
Effects of BSDZG on inflammatory cytokines in serum, including TNF-α **(A)** and IL-1β **(B)**. Representative blots and statistical analysis of the effects of BSDZG on related proteins in the prostate tissue, including TNF-α and TNFR **(C)**. ^*^ *p* < 0.05, compared with the control group; ^#^ *p* < 0.05, compared with the model group.

Pro-inflammatory cytokines, including TNF-α and IL-1β, are the prominent indicators quantifying the severity of inflammation and immune damage in the prostate tissue. In the present study, the altered tendency of the level of TNF-α and IL-1β was the same (Figure 3A, B). The expression level of TNF-α and IL-1β of the prostate tissue in the model group was significantly increased compared with the control group (*p* < 0.05). The level of TNF-α and IL-1β of the prostate tissue in the L-BSDZG group and H-BSDZG group was partially downregulated, which presented dose dependence. Furthermore, the changing trends of the expression level of proteins, TNF-α and TNFR, in the prostate tissue were also the same (Figure 3C). There was a statistical difference in the expression level of TNF-α and TNFR proteins between the control group and the model group (*p* < 0.05). After being treated by the L-BSDZG and H-BSDZG, the expression level of TNF-α and TNFR declined, among which the level of TNFR in the L-BSDZG group and the level of TNF-α and TNFR in the H-BSDZG group were dramatically decreased compared with those in the model group (*p* < 0.05).

### Effects of BSDZG on the AKT, p38 MAPK, and NF-κB pathways of the prostate tissue of rats

**FIGURE 4:**
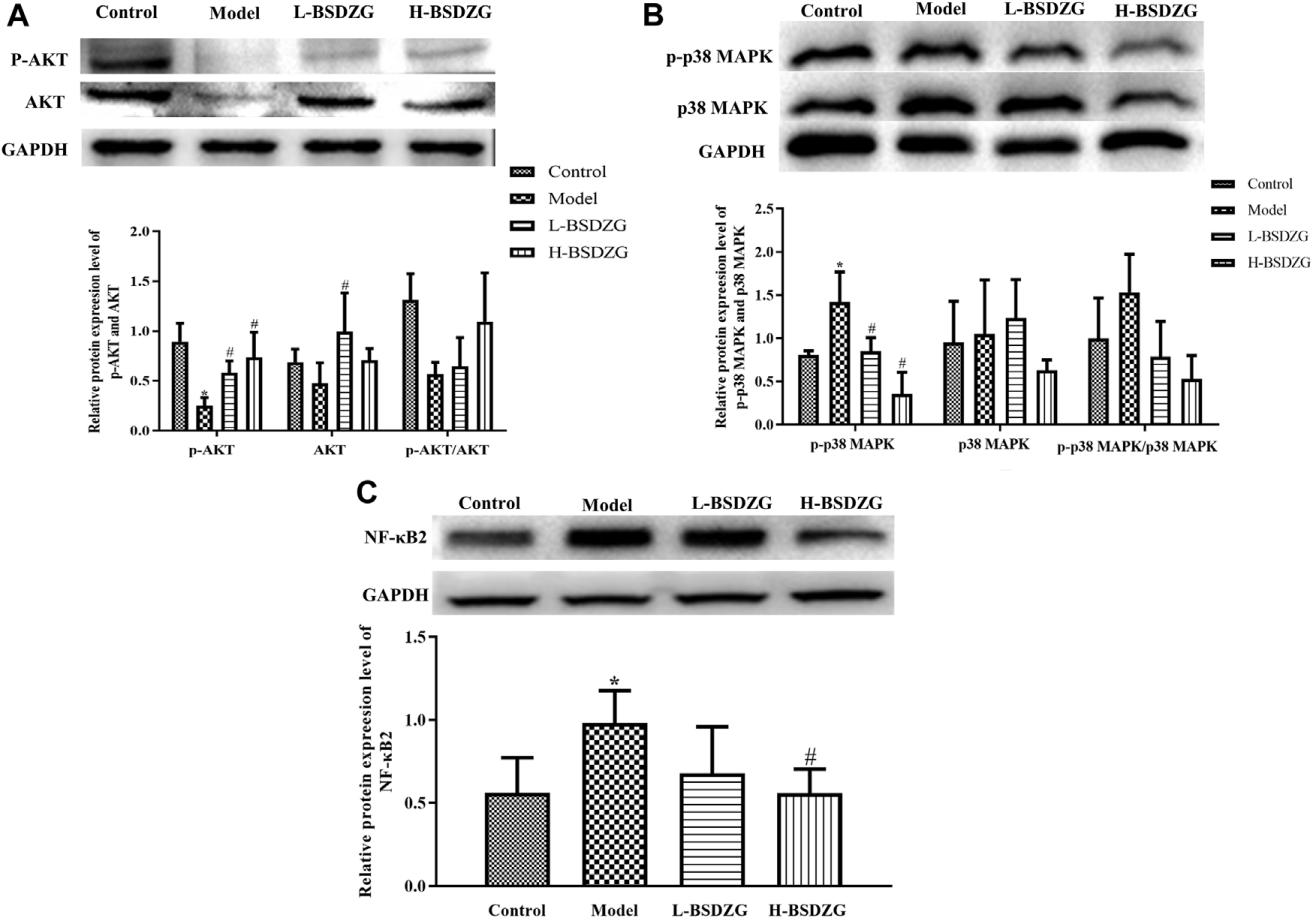
Effects of BSDZG on the protein expression level of AKT and p-AKT **(A)**, p38 MAPK and p-p38 MAPK **(B)**, as well as NF-κB2 **(C)** in the prostate tissue of rats. ^*^ *p* < 0.05, compared with the control group; ^#^ *p* < 0.05, compared with the model group.

In the present study, the expression level of AKT, p-AKT, p38 MAPK, p-p38 MAPK, and NF-κB2 in the prostate tissue was determined by adopting an approach of Western blot. As Figure 4A-C shows, compared with the control group, the expression level of p-p38 MAPK and NF-κB2 was memorably elevated but the expression level of p-AKT markedly declined in the model group (*p* < 0.05). Although the varying tendency of AKT and p38 MAPK between the control group and the model group was the same as p-AKT and p-p38 MAPK, a significant difference did not exist in both. Moreover, the expression level of p-p38 MAPK and NF-κB2 was prominently downregulated after H-BSDZG treatment, but only the prominent downregulation of p-p38 MAPK existed in the L-BSDZG group (*p* < 0.05). Although the expression level of p-AKT was substantially upregulated after administering L-BSDZG and H-BSDZG, the substantial upregulation of AKT existed in only the L-BSDZG group (*p* < 0.05).

### Effects of BSDZG on the expression level of Bax and Bcl-2 as well as the apoptosis rate of the prostate tissue of rats

**FIGURE 5:**
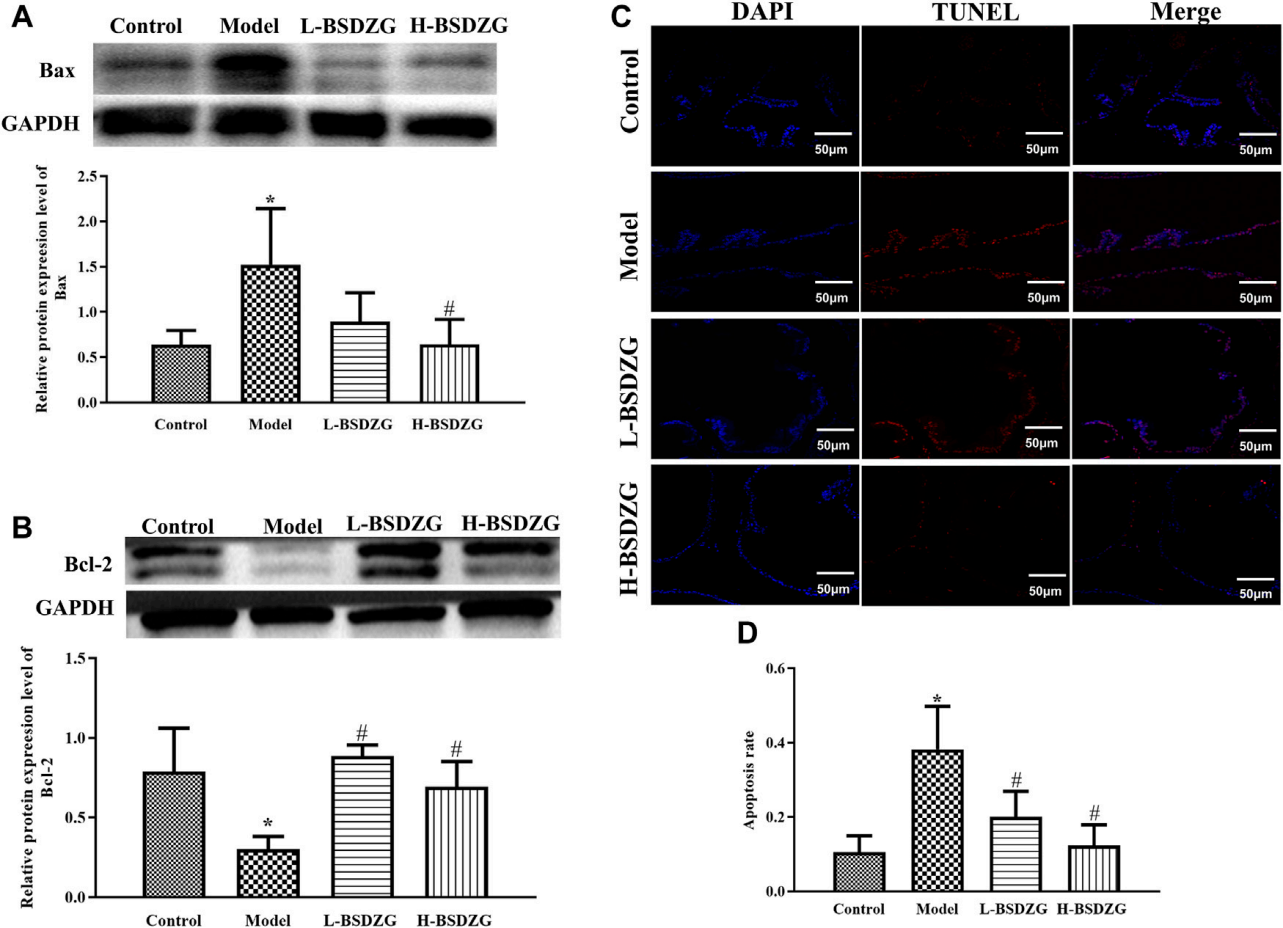
Effects of BSDZG on the protein expression level of Bax **(A)** and Bcl-2 **(B)**. Representative TUNEL staining images **(C)** and statistical analysis **(D)** of effects of BSDZG on apoptosis level of the prostate tissue. ^*^ *p* < 0.05, compared with the control group; ^#^ *p* < 0.05, compared with the model group.

Bax and Bcl-2, two significant apoptotic regulatory proteins in organisms, presented diametrically opposite changing tendencies in the present study. Illustrated in Figure 5A, B, the expression level of Bax was dramatically upregulated but the expression level of Bcl-2 was prominently downregulated after establishing the CNP model in rats (*p* < 0.05). After treatment with L-BSDZG and H-BSDZG, the expression level of Bax and Bcl-2 was reversed. The apoptosis level of the prostate tissue is an important indicator to reflect the destructive degree of chronic inflammation. As shown in Figure 5C, the number of red-stained cells of prostate epithelial tissue in both the L-BSDZG group and H-BSDZG group was higher than that in the control group but was lower than that in the model group. Moreover, the results in Figure 5D indicated that the apoptosis level in the model group was significantly elevated compared with the control group (*p* < 0.05), and the L-BSDZG and H-BSDZG both dramatically decreased the apoptosis level induced by CNP (*p* < 0.05).

### Effects of BSDZG on pro-inflammatory cytokines and related proteins in RWPE-1 cells

**FIGURE 6:**
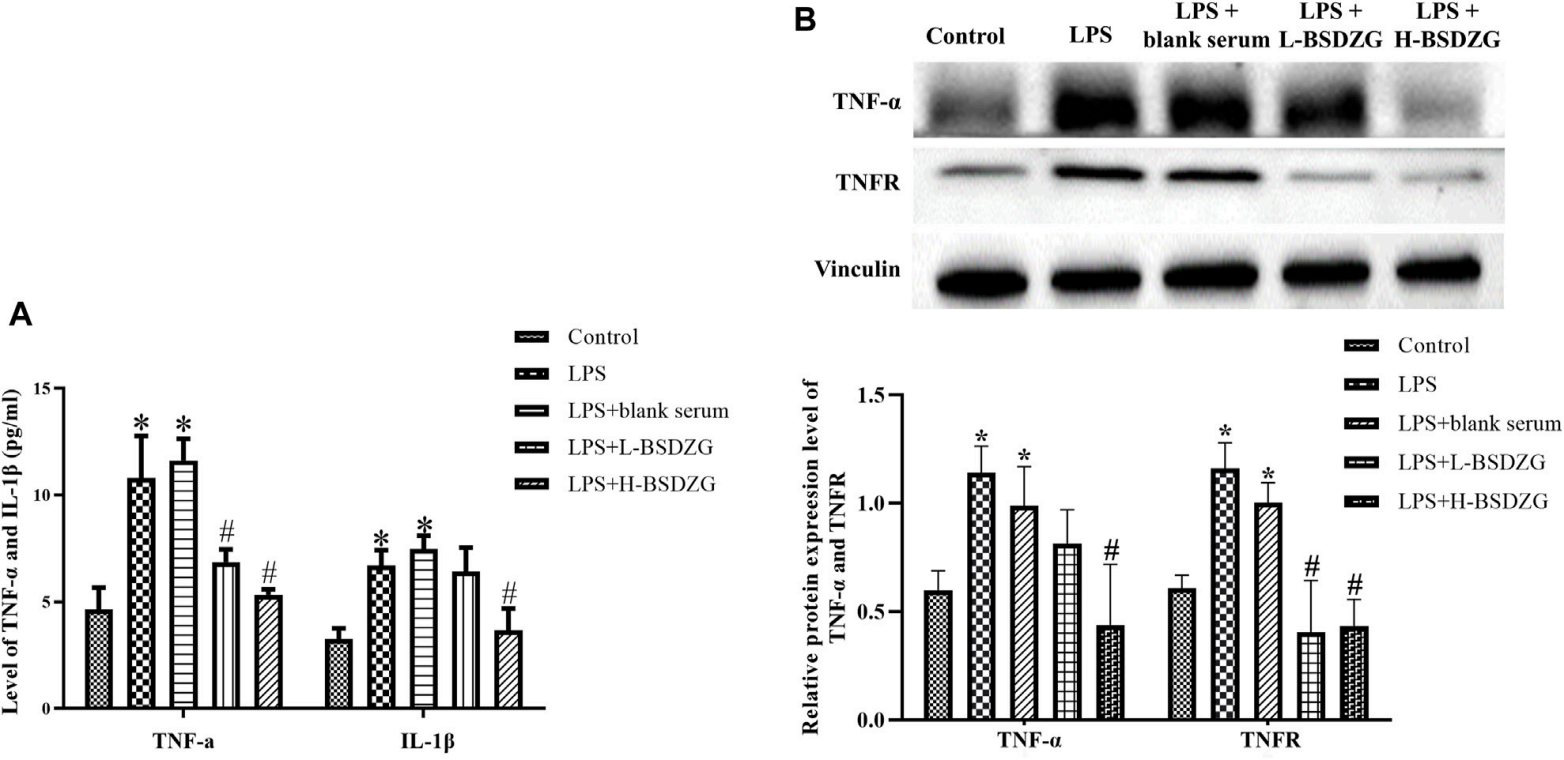
Effects of BSDZG on inflammatory cytokines in the supernatant of RWPE-1 cells, including TNF-α and IL-1β **(A)**. Representative blots and statistical analysis of the effects of BSDZG on related proteins in RWPE-1 cells, including TNF-α and TNFR **(B)**. ^*^ *p* < 0.05, compared with the control group; ^#^ *p* < 0.05, compared with the LPS + blank serum group.

The variation of the level of TNF-α and IL-1β (two dominant pro-inflammatory cytokines to regulate inflammatory responses) in the supernatant of RWPE-1 cells manifested the same tendency. As Figure 6A presented, the level of TNF-α and IL-1β in the supernatant of RWPE-1 cells was prominently increased after inducing by LPS (*p* < 0.05). After being treated with the drug-containing serum of L-BSDZG and H-BSDZG, the level of IL-1β in the LPS + H-BSDZG group and the level of TNF-α in both two groups significantly declined (*p* < 0.05). At the protein level (shown in Figure 6B), the regulatory effects of BSDZG on TNF-α and TNFR in RWPE-1 cells were the same. The expression level of TNF-α and TNFR was markedly upregulated in both LPS and LPS + blank serum groups compared with the control group (*p* < 0.05). Compared with the LPS + blank serum group, the expression level of TNF-α in the LPS + H-BSDZG group and the expression level of TNFR in the LPS + L-BSDZG and the LPS + H-BSDZG groups all substantially declined (*p* < 0.05).

### Effects of BSDZG on the AKT, p38 MAPK, and NF-κB pathways in RWPE-1 cells

**FIGURE 7:**
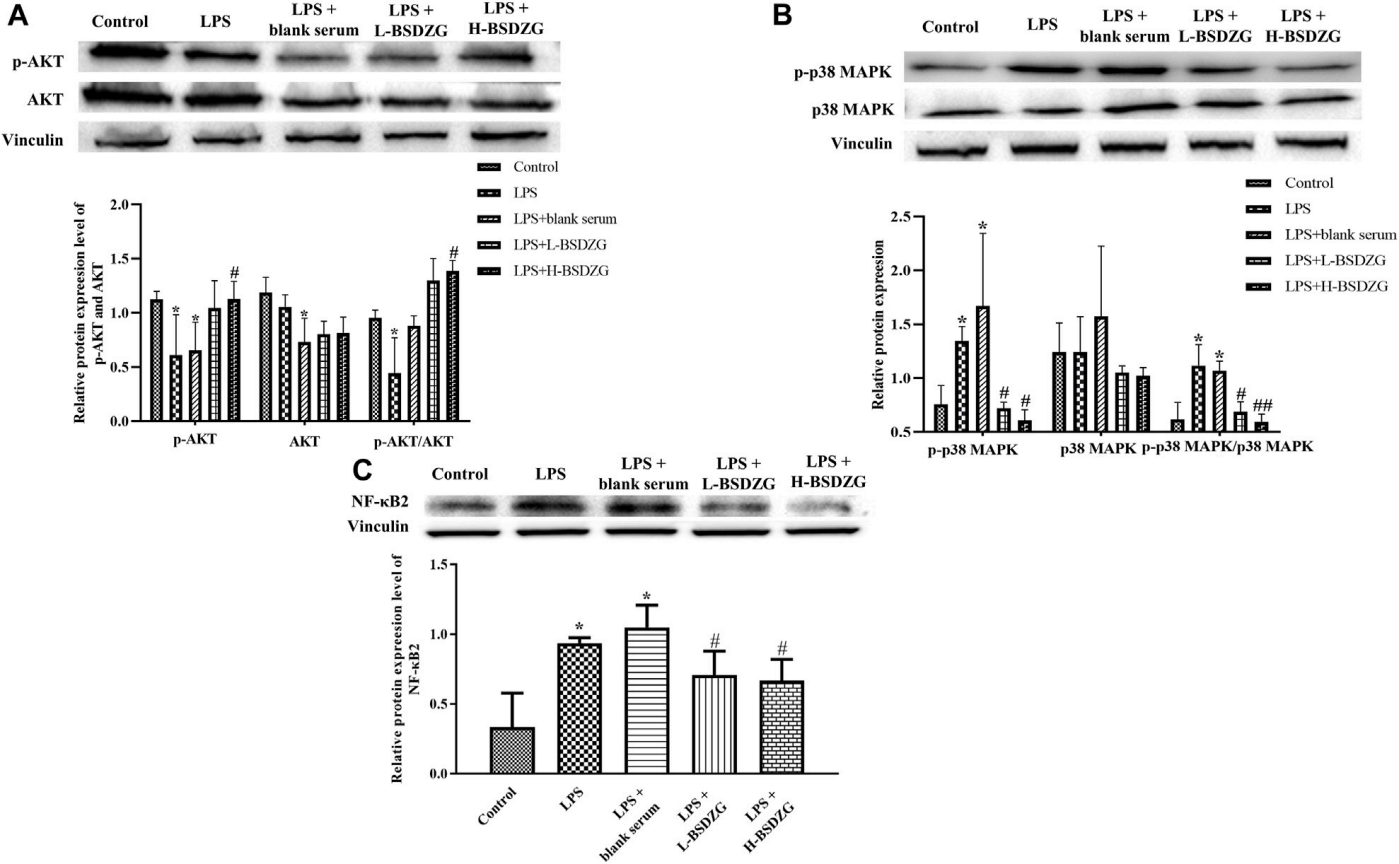
Effects of BSDZG on the protein expression level of AKT and p-AKT **(A)**, p38 MAPK and p-p38 MAPK **(B)**, as well as NF-κB2 **(C)** in RWPE-1 cells. ^*^ *p* < 0.05, compared with the control group; ^##/#^ *p* < 0.01/0.05, compared with the LPS + blank serum group.

The effects of BSDZG on the AKT, p38 MAPK, and NF-κB pathways in RWPE-1 cells were similar to those on the prostate tissue of rats. As Figure 7A-C showed, LPS led to the significant downregulation of the phosphorylation level of AKT and upregulation of the phosphorylation level of p38 MAPK and expression level of NF-κB2 in RWPE-1 cells (*p* < 0.05), and resulted in an inconspicuous modification of the expression level of AKT and p38 MAPK. After being administered by drug-containing serum of L-BSDZG and H-BSDZG, the above variations were all reversed to varying degrees, among which the phosphorylation level of AKT substantially upregulated only in the LPS + H-BSDZG group (*p* < 0.05), and the phosphorylation level of p38 MAPK as well as the expression level of NF-κB2 memorably downregulated in the LPS + L-BSDZG and LPS + H-BSDZG groups (*p* < 0.05).

### Effects of BSDZG on the expression level of Bax and Bcl-2 as well as the apoptosis rate in RWPE-1 cells.

**FIGURE 8:**
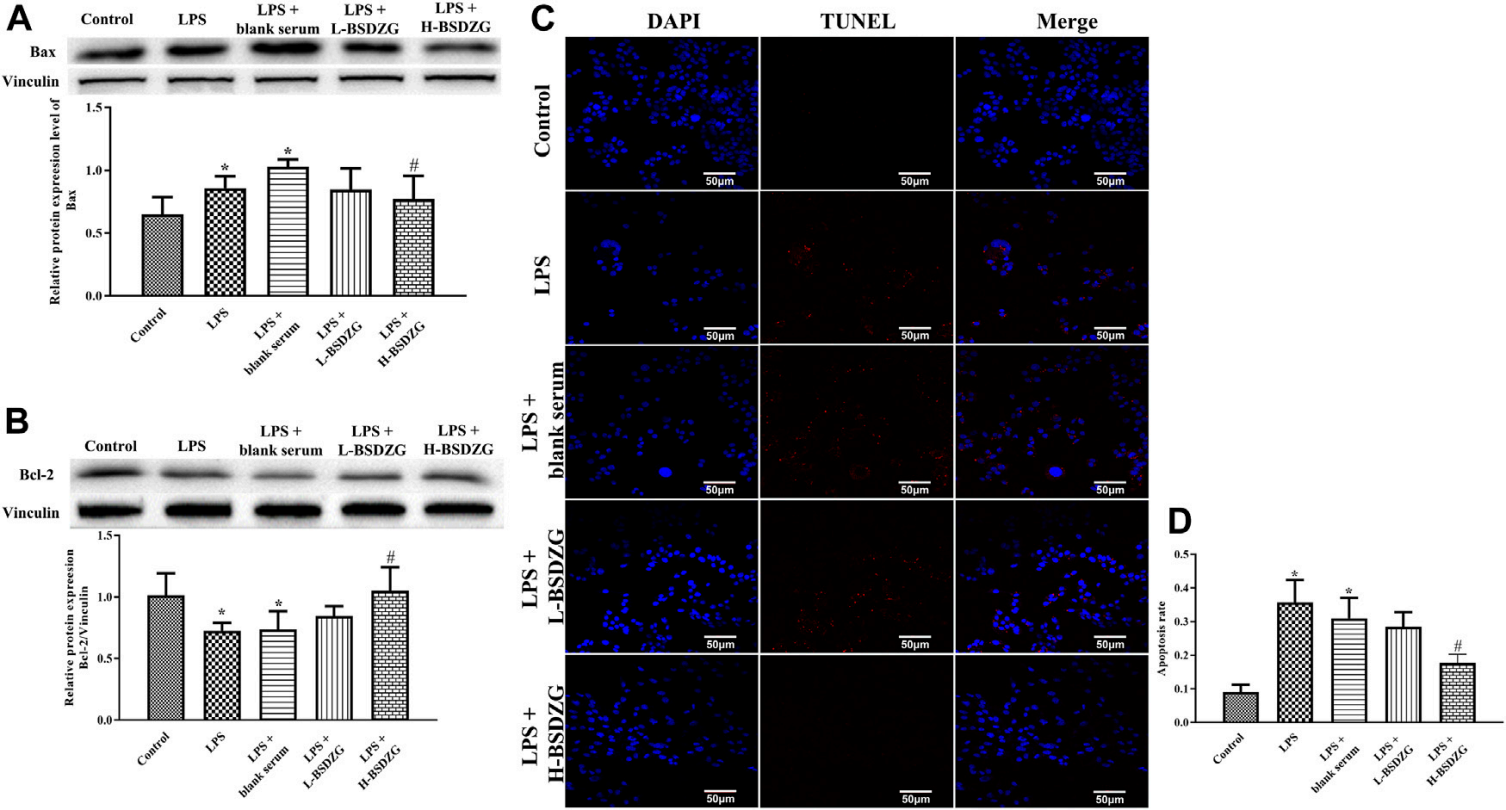
Effects of BSDZG on the protein expression level of Bax **(A)** and Bcl-2 **(B)** in RWPE-1 cells. Representative TUNEL staining images **(C)** and statistical analysis **(D)** of effects of BSDZG on apoptosis level in RWPE-1 cells. ^*^ *p* < 0.05, compared with the control group; ^#^ *p* < 0.05, compared with the LPS + blank serum group.

The effects of BSDZG on the expression level of Bax and Bcl-2 as well as the apoptosis rate in RWPE-1 cells were similar to those on the prostate tissue of rats. Illustrated in Figure 8A-D, the expression level of Bax and the apoptosis rate were prominently elevated and the expression level of Bcl-2 was markedly reduced by LPS in RWPE-1 cells (*p* < 0.05). Compared with the LPS + blank serum group, the expression level of Bax and Bcl-2 as well as the apoptosis rate were dramatically reversed only in the LPS + H-BSDZG group (*p* < 0.05), but not in the LPS + L-BSDZG group.

### Effects of overexpression of p38 MAPK on pro-inflammatory cytokines in RWPE-1 cells

**FIGURE 9:**
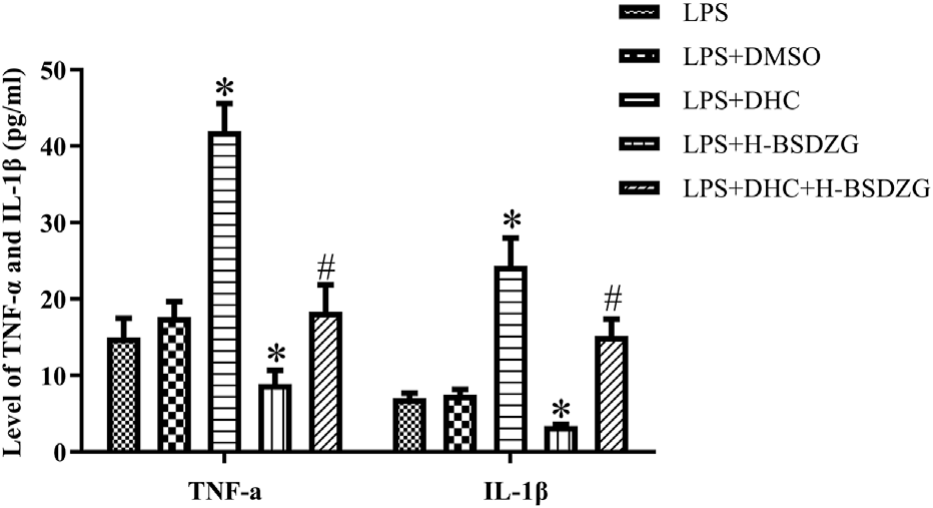
Effects of overexpression of p38 MAPK on inflammatory cytokines in the supernatant of RWPE-1 cells, including TNF-α and IL-1β. ^*^ *p* < 0.05, compared with the LPS group; ^#^ *p* < 0.05, compared with the LPS + DHC group.

As the above experiments’ results demonstrated, H-BSDZG was expected to exert anti-apoptotic and anti-inflammatory effects on RWPE-1 cells induced by LPS via regulating the AKT, p38 MAPK, and NF-κB pathways. Therefore, the protein of p38 MAPK was overexpressed to further investigate the interaction mechanism between the anti-inflammatory and anti-apoptotic effects of H-BSDZG. As Figure 9 shows, overexpression of p38 MAPK memorably upregulated the level of two pro-inflammatory cytokines (i.e., TNF-α and IL-1β) in RWPE-1 cells (*p* < 0.05). Moreover, the level of TNF-α and IL-1β in the LPS + DHC + H-BSDZG group was lower than those in the LPS + DHC group (*p* < 0.05). The above results indicated that the anti-inflammatory effect of BSDZG was closely connected with its anti-apoptotic effect.

### Effects of overexpression of p38 MAPK on p38 MAPK and NF-κB pathways in RWPE-1 cells

**FIGURE 10:**
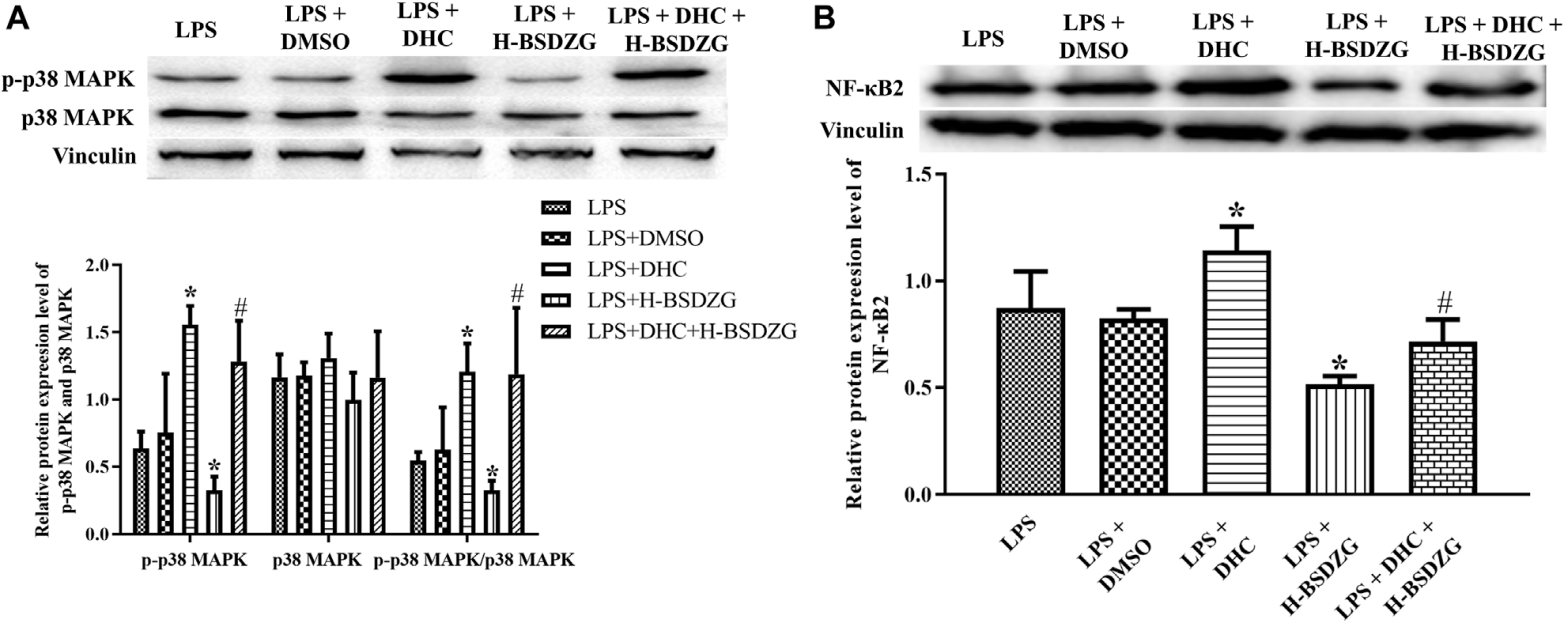
Effects of overexpression of p38 MAPK on the protein expression level of p-p38 MAPK and p38 MAPK **(A)**, as well as NF-κB2 **(B)** in RWPE-1 cells. ^*^ *p* < 0.05, compared with the LPS group; ^#^ *p* < 0.05, compared with the LPS + DHC group.

As Figure 10A, B presented, the expression level of p-p38 MAPK and NF-κB2 as well as the level of p-p38 MAPK/p38 MAPK were prominently upregulated when treating RWPE-1 cells with DHC (p38 MAPK activator). Compared with the LPS + DHC group, the expression of p-p38 MAPK and NF-κB2 as well as the level of p-p38 MAPK/p38 MAPK were dramatically downregulated in the LPS + DHC + H-BSDZG group. The anti-inflammatory effect of H-BSDZG, achieved by inhibiting the p38 MAPK pathway to suppress the NF-κB pathway, on RWPE-1 cells induced by LPS was further confirmed by overexpressing the p38 MAPK.

## Discussion

CNP, one of the most common chronic diseases in urology, not only leads to the fibrosis of prostates but also tends to induce sexual dysfunction and emotional abnormalities due to the characteristics of unknown etiology and easy recurrence, as well as the insufficient understanding of it from patients, which seriously endangers the physical and mental health of humanity (Lipsky, Byren et al., 2010). It was suggested that the balance of bacterial community in the prostate and low urethra tract plays an important role in the prevention and treatment of CNP (Hou, Long et al., 2012). The autoimmune phenomenon formed by the proliferative response of T cells to seminal plasma proteins was also one significant inducement of CNP (Batstone, Doble et al., 2002). It was also demonstrated that the endocrine abnormalities caused by the dysfunctional hypothalamic-pituitary-adrenal axis, especially for the enhancement of awakening cortisol responses, tended to induce CNP (Anderson, Orenberg et al., 2008). Moreover, it was discovered that the occurrence of some clinical characteristics, such as urine reflux, epithelial dysfunction in the lower urinary tract, and epithelial dysfunction in the lower urinary tract, increased the morbid risk of CNP (Dellabella, Milanese et al., 2006; Parsons, 2007; Shoskes, Berger et al., 2008). In addition, although it is widely acknowledged that inflammatory response and oxidative stress are the dominant pathological mechanisms of CNP, the involved regulatory pathways are diverse (Paulis, 2018). As far as inflammatory response, the regulation of various pathways, such as the NLRP3 inflammasome-mediated Caspase-1/GSDMD pyroptosis pathway and the NF-κB pathway, effectively mitigates chronic prostatitis (Zhao, Yang et al., 2020; Liu, Guo et al., 2023). For oxidative stress, the suppression of ferroptosis induced by lipid peroxidation and the regulation of the typical antioxidant pathway, PI3K/AKT/FOXO1, contribute to alleviating pathological injuries of chronic prostatitis (Feng, Dong et al., 2021; Lin, Zhang et al., 2022). Due to the complicated pathological mechanism of CNP, the complete treatment plan for CNP has not been established yet, and the existing treatment principles for CNP are focused on mitigating clinical symptoms, such as pain, dysuria, as well as mental and psychological disorders (Zang, Tian et al., 2021). Although the clinical drugs contribute to treating CNP, their low efficiency and adverse reactions (e.g., alpha-blockers, M-receptor blockers, nonsteroidal anti-inflammatory drugs, 5 alpha-reductase inhibitors, and antibiotics) severely restrict their application (Lü, Zhao et al., 2004). It is urgent to investigate one therapy to effectively cure CNP with low adverse reactions. Out of the characteristics of multi-component and multi-targets, traditional Chinese medicine possesses a bright application prospect in treating CNP. Therefore, in the present study, we aimed to investigate the novel therapeutic mechanism of BSDZG that is extensively adopted for the clinical therapy of CNP in China on curing CNP.

Although the pathological mechanism of CNP is intricate, the previous studies affirmed that anti-inflammation and anti-apoptosis were the two primary therapeutic mechanisms. It was confirmed that tadalafil effectively mitigated the inflammatory reaction and oxidative stress injury induced by LPS in RWPE-1 cells by regulating the Akt/Nrf2 pathway (Song, Chen et al., 2021). Moreover, several studies also demonstrated the Akt/mTOR signaling pathway was a promising therapeutic candidate for chronic prostatitis (Zhan, Chen et al., 2020; Zhang, Yu et al., 2020). The communal protein from the above two signaling pathways, Akt, is closely related to the growth, proliferation, and apoptosis of cells, as well as inflammatory response, angiogenesis, and tumor development (Linton, Moslehi et al., 2019). The MAPK signaling pathway, especially the p38 MAPK pathway, mediates the generation and release of multiple inflammatory cytokines by participating in the regulation of various inflammatory mediators (Yeung, Aziz et al., 2018). Some studies also proved that the suppression of p38 MAPK and NF-κB pathways contributed to alleviating CNP from the perspectives of bioinformatics analysis and experimental research (Yang, Meng et al., 2019; Zhang, Liu et al., 2019). As a nuclear protein factor, NF-κB plays a central transcriptional regulatory role in diverse genes and participates in regulating various inflammatory cytokines to exert inflammatory and immune regulatory functions (Yu, Lin et al., 2020). In the prostate, NF-κB that was activated by pro-inflammatory cytokines, TNF-α, substantially resulted in immune disorders and inflammatory reactions, which play a monumental role in the pathogenesis of chronic prostatitis (Hu, Yang et al., 2016). Hence, in the present study, we investigated the therapeutic effects of BSDZG on CNP from the aspects of anti-inflammation and anti-apoptosis *in vivo* and *in vitro* and explored the interaction between the p38 MAPK and NF-κB pathway based on the mechanism research of BSDZG on treating RWPE-1 cells induced by LPS *in vitro*.

*In vivo*, the experimental results proved that BSDZG was able to decrease the prostate index and alleviate the pathological injury of the prostate tissue induced by CNP at the macro level. Moreover, at the micro level, the *in vivo* and *in vitro* experimental results demonstrated that BSDZG also declined the apoptosis rate, the level of pro-inflammatory cytokines (i.e., IL-1β and TNF-α), the expression level of related proteins (e.g., Bax, TNF-α, TNFR, p-p38 MAPK, and NF-κB2), and upregulated the expression level of Bcl-2 and p-AKT. In addition, although BSDZG reversed the level of p-AKT/AKT and p-p38 MAPK/p38 MAPK both *in vivo* and *in vitro*, there are significant differences between groups only *in vitro* and not *in vivo*. We speculated that it may be caused by the biological differences between rats. The above results indicated that BSDZG exerts anti-apoptotic and anti-inflammatory effects in treating CNP via declining the level of pro-inflammatory cytokines TNF-α and related protein TNFR to regulate the AKT, p38 MAPK, and NF-κB signaling pathways *in vivo* and *in vitro*. Then, the p38 MAPK was hyperphosphorylated to further investigate the interaction between the p38 MAPK and NF-κB pathway based on the mechanism research of BSDZG on treating RWPE-1 cells induced by LPS *in vitro*. The experimental results suggested that DHC not only activated the expression of p-p38 MAPK but also enhanced the expression level of NF-κB2 and the level of IL-1β and TNF-α. The high dose of BSDZG effectively reversed the above alterations caused by DHC. Therefore, we considered that the anti-inflammatory effect of BSDZG in curing CNP was embodied in inhibiting the p38 MAPK pathway to suppress the NF-κB pathway.

**FIGURE 11:**
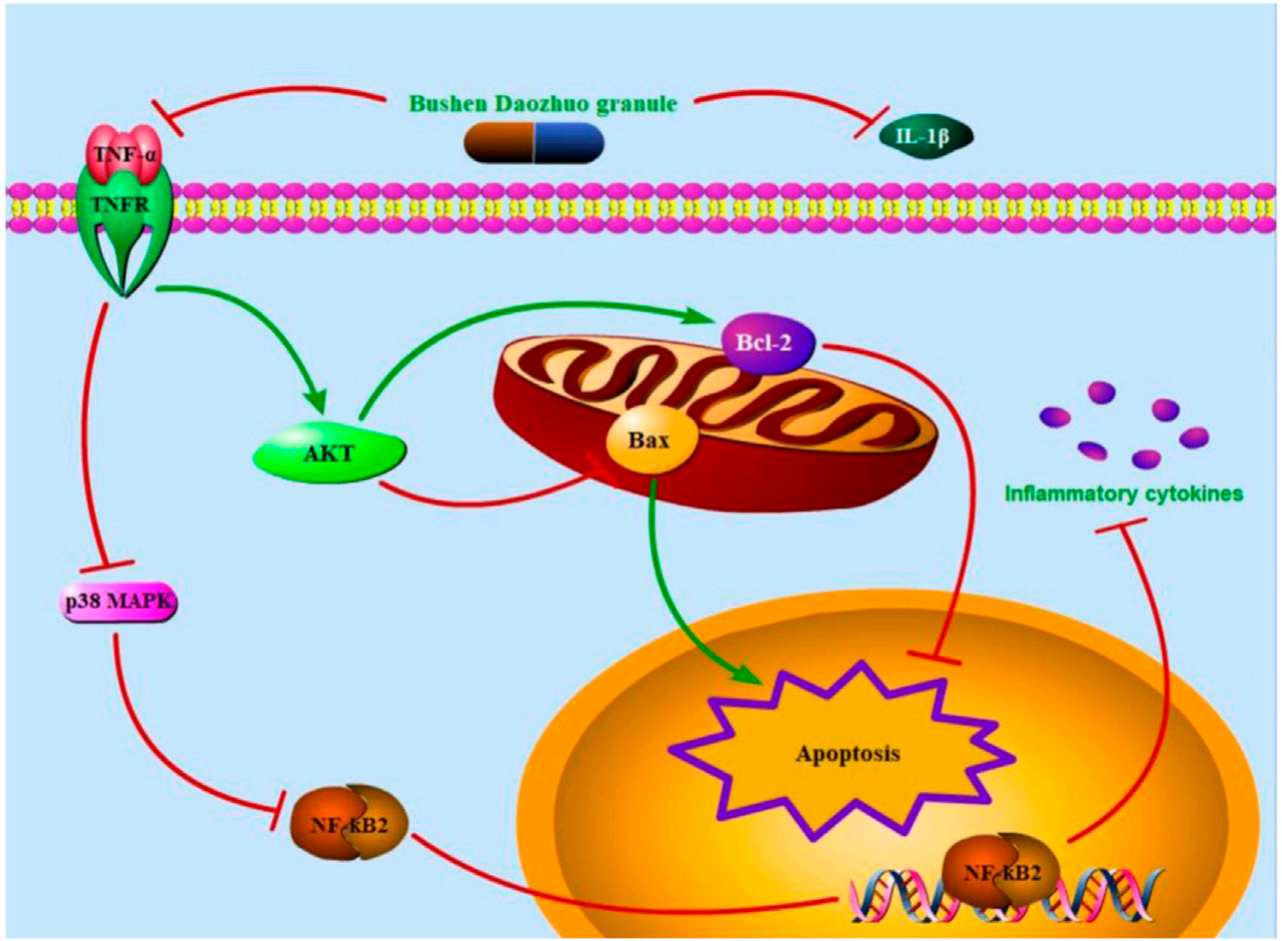
The therapeutic mechanism of BSDZG in treating CNP. The extracellular level of TNF-α and IL-1β was first declined by BSDZG. The protein expression level of TNFR was subsequently depressed to prevent the entrance of TNF-α into the intracellular. On one hand, the AKT signaling pathway was activated to keep the expression balance of Bax and Bcl-2, exerting anti-apoptosis. On the other hand, the p38 MAPK pathway was inhibited to suppress the downstream NF-κB pathway to prevent the further release of inflammatory cytokines, exerting anti-inflammation.

Taken together, we attested that one of the *in vivo* and *in vitro* therapeutic mechanisms of BSDZG on CNP were reflected as the anti-inflammation and anti-apoptosis that was formed by suppressing the level of pro-inflammatory cytokines, TNF-α and IL-1β, to regulate the AKT, p38 MAPK, and NF-κB pathways (Figure 11), among which the anti-inflammatory effect of BSDZG was realized by suppressing the p38 MAPK pathway to inhibit the downstream NF-κB pathway. However, the other possible mechanisms of BSDZG in treating CNP are inevitably ignored because the interests of this research were mainly focused on anti-inflammation and anti-apoptosis. In recent years, several studies reported that the imbalance of gut microbiota played a crucial role in the occurrence and development of CNP, which has become a hotpot in the diagnosis, treatment, and prevention of CNP (Liu, Yu et al., 2020; Yu, Hu et al., 2022). Therefore, the specific mechanism of BSDZG based on the gut microbiota in the treatment of CNP remains to be further explored in the future.

## Data Availability

The original contributions presented in the study are included in the article/Supplementary Material, further inquiries can be directed to the corresponding author.

## Abbreviations

AKT, protein kinase B; BCA, bicinchoninic acid; BS, blank serum; BSDZG, Bushen Daozhuo granule; CNP, chronic non-bacterial prostatitis; DHC, dehydrocorydaline; DMSO, Dimethyl sulfoxide; FBS, fetal bovine serum; H-BSDZG, high dose of BSDZG; i.g, oral gavage; i.h, hypodermic injection; IL-1β, interleukin-1β; L-BSDZG, low dose of BSDZG; LPS, lipopolysaccharide; NF-κB, nuclear factor kappa-B; p-AKT, phosphorylated AKT; p38 MAPK, p38 mitogen-activated protein kinase; p-p38 MAPK, phosphorylated p38 MAPK; TNF-α, tumor necrosis factor-α; TNFR, tumor necrosis factor receptor.
