# Supplementary material for: The Protective Effects of Bushen Daozhuo Granule on Chronic Non-bacterial Prostatitis
Source: Front Pharmacol. 2024 Jan 4;14:1281002. doi: 10.3389/fphar.2023.1281002 (PMC10794918; doi:10.3389/fphar.2023.1281002)
Supplement: Supplementary file 1 [file Table1.DOCX]

| Number | Name |
| --- | --- |
| 1 | *Cuscuta chinensis* Lam. |
| 2 | *Dioscorea septemloba* Thunb. |
| 3 | *Schisandra chinensis* (Turcz.) Baill. |
| 4 | *Plantago asiatica* L. |
| 5 | *Alpinia oxyphylla*Fruit. |
| 6 | *Lindera aggregata* (Sims) Kosterm. |
| 7 | Concha Ostreae |
| 8 | *Acorus tatarinowii* Schott. |
| 9 | *Verbena officinalis* L. |
| 10 | *Hirudo* Linnaeus |
| 11 | *Saponaria vaccaria*L. |

**Supplementary table 1.** The list of eleven kinds of Chinese materia medica in Bushen Daozhuo granule.
